# Effects of milk replacer feeding level on growth performance, rumen development and the ruminal bacterial community in lambs

**DOI:** 10.3389/fmicb.2022.1069964

**Published:** 2023-01-10

**Authors:** Yongliang Huang, Guoxiu Wang, Qian Zhang, Zhanyu Chen, Chong Li, Weimin Wang, Xiaoxue Zhang, Xiaojuan Wang, Deyin Zhang, Panpan Cui, Zongwu Ma

**Affiliations:** ^1^College of Animal Science and Technology, Gansu Agricultural University, Lanzhou, China; ^2^State Key Laboratory of Grassland Agro-Ecosystems, College of Pastoral Agriculture Science and Technology, Lanzhou University, Lanzhou, China

**Keywords:** lambs, milk replacer, rumen development, rumen fermentation, fiber degradation, rumen microbiota

## Abstract

Feeding with a suitable level of milk replacer (MR) can improve the survival rate and stimulate the growth potential of early lambs. However, feeding excessive MR might be detrimental to rumen development and microbial colonization. Herein, we investigated the effects of feeding different levels of MR on rumen digestive function and ruminal microorganisms. Fourteen healthy male Hu lambs with similar birth weights and detailed pedigree records were divided into two groups to receive low (2% of average body weight per day) and high (4% of average body weight per day) levels of MR. We analyzed the effects of the MR feeding level on growth performance, fiber degradation rates, rumen fermentation parameters, enzyme activities and rumen histomorphology. We found that feeding with a high level of MR improved the average daily gain of early lambs, but decreased the starter intake, rumen weight and papillae length. We also analyzed the effects of the MR feeding level on the rumen microbiota using 16S-rRNA amplicon sequencing data. The results showed that high a MR feeding level increased the rumen microbial diversity but decreased the abundance of many carbohydrate degrading bacteria. Several bacterial genera with significant differences correlated positively with rumen cellulase activity and the acid detergent fiber degradation rate. Our results suggested that a high level of MR could improve the growth performance of early lambs in the short term; however, in the long term, it would be detrimental to rumen development and have adverse effects on the adaptation process of the microbiota to solid feed.

## 1. Introduction

Artificial rearing of lambs using milk replacer (MR) is widely applied in sheep husbandry because of insufficient milk secretion of ewes, mastitis, postpartum paralysis and other reasons (Belanche et al., 2019; McCoard et al., 2020; Arshad et al., 2021; Mialon et al., 2021). Artificial feeding to make up for the lack of breast milk can increase the survival rate of lambs, shorten the breeding cycle of ewes, and improve animal welfare. In China, Hu sheep constitute an important livestock for lamb production because of their excellent prolificacy, rapid growth, and ability to adapt to poor-quality feeds and diverse environments (Yue, 1996; He et al., 2019). However, multiparous ewes also easily lead to insufficient breastmilk for lambs. Artificial rearing is important for the utilization of surplus lambs from dairy sheep systems, and lambs from meat sheep operations that are not able to be naturally reared to generate additional revenue from meat production (McCoard et al., 2020). Therefore, the rational utilization of MR significant to improve the survival rate and growth performance of lambs.

MR is an artificial milk produced by replacing milk protein with non-milk protein based on the nutritional standards of breast milk (Li et al., 2021). The nutritional components and physical form of MR are similar to breast milk, and its quality is not easily affected by the external environment (Toral et al., 2015). Evidence suggests that feeding lambs with MR could accelerate growth performance, has long-term benefits for sheep health (Zhang et al., 2019), enhance immunity, reduce the stress reaction caused by sudden changes of diets (Amdi et al., 2021), and affect post-weaning starter intake and average daily gain (Chapman et al., 2016). The beneficial effects of MR administration in early-weaning lambs also function *via* regulating their rumen microbiota (Bhatt et al., 2009).

However, high levels of MR might be detrimental to rumen development in early lambs and affect their rumen microbiota. The rumen of a newborn ruminant is not completely developed and does not contain a fully established microflora (Ekiz et al., 2016); early lambs rumen function is similar to monogastric animals (Longenbach and Heinrichs, 1998). The period from birth to 2 months of age is a critical stage of rumen development, representing a time window to regulate microbial colonization in lambs, and the feeding strategy in the first few weeks of life has been reported to affect rumen development (Carballo et al., 2019). Although little research has been conducted on the effects of MR feeding level on the rumen development, excessive intake of MR might reduce the intake of starter feed, which is essential for rumen development. Reports suggested that feeding high levels of MR could reduce post-weaning starter digestibility, especially for NDF (neutral detergent fiber) and ADF (acid detergent fiber; Terré et al., 2006; Hill et al., 2010). Low digestibility in young ruminants fed large quantities of MR is likely to be associated with suboptimal development of the rumen (Hill et al., 2016). These changes might be closely related to the rumen microbiota. Furman et al. proposed that both deterministic effects, driven by age and diet, and stochastic effects, driven by early colonization events, shape the composition of the rumen microbiome throughout life (Furman et al., 2020). MR is usually fed quantitatively in artificially reared lambs, and the feeding level varies greatly in different farms. Therefore, it is important to clarify the effect of the MR level on the rumen microbiota and its relationship with lamb development, and to balance rumen development and microbiota colonization using an appropriate feeding MR level. In recent years, several studies have examined the length of MR feeding period on rumen fermentation and microbial diversity (Carballo et al., 2019; Zhang et al., 2019; Mao et al., 2021), but relatively few have been directed at assessing how MR feeding level can affect rumen development. The appropriate MR level for lambs and the effect of feeding MR on rumen development and microbial colonization remains unclear.

Thus, in the present study, we hypothesized that feeding early lambs with intensive MR would affect rumen function and the rumen microbiota through changes in feed structure and intake, thereby affecting performance. We analyzed the growth performance and starter intake of lambs fed with different levels of MR, and evaluated the development and functionality of the rumen *via* ruminal fiber degradation rates, rumen weight, fermentation parameters, enzyme activities and rumen histomorphology. Furthermore, we employed 16S rRNA sequencing to explore the effect of the MR feeding level on the rumen microbiota and its relationship with rumen function and development. A detailed understanding of the regulation of early rumen development (function, morphology, and colonization) could provide the basis for the rational use of early nutritional regulation strategies to improve the productivity and health of lambs.

## 2. Materials and methods

### 2.1. Experimental design and animal handling

The experimental animals were selected from a commercial sheep farm (Minqin Zhongtian Sheep Industry Co. Ltd., Gansu, China), and 14 Hu male lambs with detailed pedigree records were randomly divided into two groups according to the principle of similar body weight (mean ± SD: 3.29 ± 0.68 kg). They were divided into the low MR feeding level group (L, 2% of average body weight per day). The L group received a traditional MR feeding quantity, which was 2% of average body weight per day, following the feeding guidelines of producer (Beijing Precision Animal Nutrition Research Center, Beijing, China; Yue et al., 2011). The H group received an intensive MR feeding quantity at 4% of average body weight per day, which has been reported to have a great impact on the growth performance of early lambs (Zhang et al., 2019). The MR contains 96.91% dry matter (DM), 23.22% protein, and 13.20% fat. All lambs were kept indoors with ewes to ensure adequate colostrum intake for 3 days after birth, from 3 days old for training to consuming MR, and to 7 days old to completely replace breast milk with MR and start feeding with the same starter. The daily MR was subdivided in three parts, dissolved in five times the weight of warm water (40 ± 1°C) and was artificially fed at 09:00, 15:00, and 21:00. All experimental lambs were reared in single cages (1.2 m × 1 m × 1 m; 1.2 m^3^) with free access to the diet [the formula of the diet met the requirements of the Standards for Feeding Sheep and Goats for Meat issued by China (NYT816-2004)] and water. The diet formula and nutritional composition are shown in Table 1 (Huang et al., 2022). The experimental lambs were slaughtered and sampled at 49 days old.

**Table 1:** Ingredients and chemical composition of starter diet and milk replacer (air-dried basis).

Items	Starter^1^	Milk replacer
Ingredients [%]		
Alfalfa meal	18.50	
Corn	21.00	
Extruded corn	22.30	
Bran	6.00	
Soybean meal	21.50	
Extruded soybean	4.00	
Corn gluten meal	5.00	
Limestone	0.30	
Premix^2^	1.00	
NaCl	0.40	
Total	100.00	
Chemical composition		
DM (%)	90.96	96.91
DE (MJ·kg^−1^)	13.01	/
CP (%)	19.50	23.22
Fat (%)	1.33	13.20
Starch (%)	33.10	0.00
NDF (%)	18.87	0.00
ADF (%)	8.60	0.00

^1^The starter was pelleted. ^2^Premix provided the following per kg of the starter: 25 mg Fe as FeSO_4_⋅H_2_O; 40 mg Zn as ZnSO_4_⋅H_2_O; 8 mg Cu as CuSO_4_⋅5H_2_O; 40 mg Mn as MnSO_4_⋅H_2_O; 0.3 mg I as KI; 0.2 mg Se as Na_2_SeO_3_; 0.1 mg Co as CoCl_2_; 940 IU vitamin A;111 IU vitamin D;20 IU vitamin E, and; 0.02 mg vitamin B12.

### 2.2. Measurement of growth performance and starter diet intake

We first examined the effect of the MR feeding level on the growth performance and starter intake of lambs. All lambs were weighed at birth and then weighed every 7 days to calculate the average body weight and average daily gain, and to adjust the MR feeding scale. Starting from 7 days old, the starter intake of each lamb was recorded daily as the difference between offered and refused feed, and the average intake was calculated.

### 2.3. Sample collection

All the experimental lambs were slaughtered at 49 days old after a fasting period of 12 h. After the rumen contents were mixed evenly, the rumen pH was measured immediately using an acidity meter (Sartorius PB-10, Sartorius Biotech Inc., Gottingen, Germany), the mixed rumen contents were collected in sterile tubes and stored at-80°C for rumen microbial and fiber degradation rate analysis. The contents were filtered through four layers of sterilized medical gauze, packed in cryovials, and stored in-20°C refrigerator for ruminal fermentation and enzymic activity analysis. After sampling, the rumen was weighed, segments of the ruminal tissue were collected at the location of cranial ventral sac and fixed in 4% paraformaldehyde for morphology measurements.

### 2.4. Measurement of the rumen fiber degradation rate

To better understand how the MR feeding level affected rumen function in lambs, we examined the ruminal NDF and ADF degradation rates after a 12 h fast. The rumen degradation rates of neutral detergent fiber (NDF) and acid detergent fiber (ADF) after 12 h of fasting were determined using the acid-insoluble ash method (Keulen and Young, 1977). The rumen contents and starter were dried at 65°C for 8 h, and then the contents of NDF, ADF, and acid-insoluble ash were determined, respectively. The NDF and ADF were determined following the method of Van Soest et al. (1991), and acid infusible ash was determined according to the method reported by Soltani et al. (2020). The degradation rates of NDF and ADF were evaluated using the acid-insoluble ash as internal markers and were calculated according to the indirect digestibility method (Keulen and Young, 1977). The calculation of the NDF or ADF degradation rate was as follows:

NDF or ADF degradation rate (%) = [1−(A/B) × (FB/FA)] × 100,

where A was the acid-insoluble ash concentrations in the starter, B was the acid-insoluble ash concentrations in the rumen contents, FA was the NDF or ADF concentrations in the starter, and FB was the NDF or ADF concentrations in the rumen contents.

### 2.5. Measurement of the rumen metabolic phenotypes and digestive enzymatic activity

Determination of volatile fatty acids (VFA) used metaphosphorylated rumen fluid and a gas chromatographic method (Zhang Y. et al., 2021). For VFA determination, the supernatant was carefully collected and filtered through a 0.45-μm syringe filter. The clear supernatant was transferred to a vial for gas chromatography (GC). The VFA values were determined using a TRACE-1300 series GC ultra-gas chromatograph (Thermo Scientific, Milan, Italy). Total ruminal nitrogen, ammonia nitrogen, urea nitrogen, the enzymatic activity of cellulase and protease were determined using commercial assay kits (Jiancheng Bioengineering Institute, Nanjing, China) according to the manufacturer’s instructions.

### 2.6. Measurement of histomorphology in the rumen

**Figure 1:**
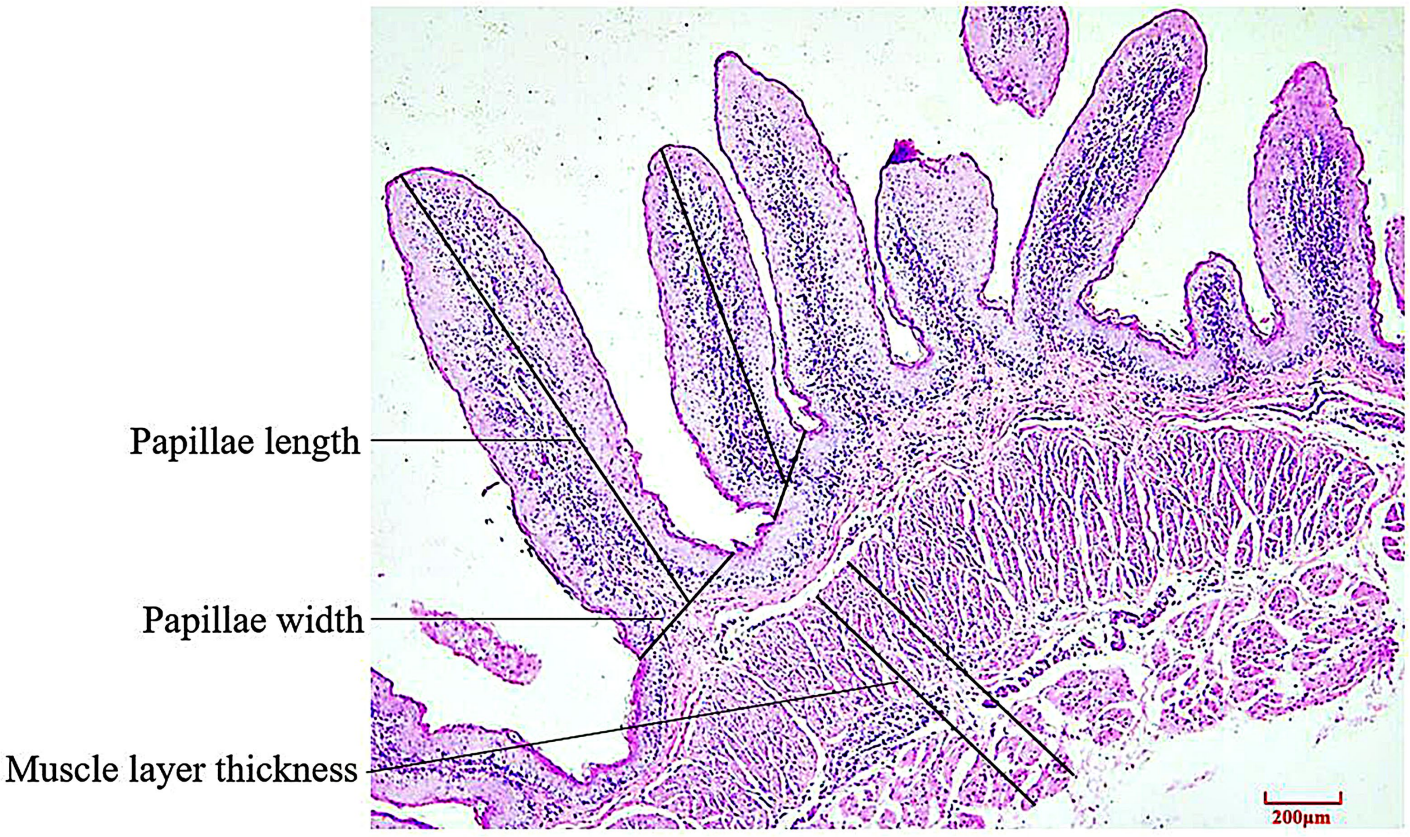
Schematic diagram of histological sections and determination of rumen tissue (HE staining).

Rumen specimens were embedded in paraffin, sectioned and stained with hematoxylin–eosin. In triplicate, 5 intact well-oriented papillae were selected for each ruminal cross section. Papillae length, papillae width and muscle layer thickness were determined using an image analysis system (Motic Image Plus 2.0, Motic China Group Co. Ltd., Xiamen, China). Papillae length was measured from the apex to the base of the papilla along its axis, papillae width was measured at bottom of papillae height and muscle layer thickness was measured from the junction between the submucosal and muscular layers to that between the muscular layer and the tunica serosa (Figure 1).

### 2.7. DNA extraction and high-throughput sequencing

Total DNA of rumen contents was extracted using an Omega E.Z.N.A. Stoll DNA kit (Omega Bio-Tek, Winooski, VT, United States). The sequencing process was the same as that detailed our previous study (Li et al., 2022). DNA quality and quantity were assessed using a NanoDrop 2000 spectrophotometer (NanoDrop Technologies Inc., Wilmington, DE, United States). The DNA was diluted to 50 ng/μl and amplicons were prepared for high throughput sequencing. PCR was used to amplify the V3–V4 regions of the 16S rRNA gene using the universal primers 341-F (50-CCTAYGGGRBGCASCAG-30) and 806-R (50-GGACTACNNGGGTATCTAAT-30). The reactions consisted of an initial incubation at 98°C for 3 min; followed by 21 cycles of 95°C for 30 s, 55°C for 45 s, and 72°C for 1 min; and a final extension step at 72°C for 7 min. Bar-coded amplicons were mixed at equimolar ratios, used for Illumina paired-end library preparation and cluster generation, and sequenced on an Illumina Hiseq2500 instrument (San Diego, CA, United States) to generate 250 bp paired-end reads.

### 2.8. Sequence and statistical analysis

Paired-end reads were merged using FLASH (V1.2.7),^1^ and the merged sequences were termed raw tags. The raw tags were filtered through a quality control pipeline using the Quantitative Insight into Microbial Ecology (QIIME) tool kit. The effective tags were assigned to operational taxonomic units (OTUs) with a 97% identity threshold using the Uparse (v7.0.1001),^2^ and taxonomy was assigned using SILVA^3^ in mothur with a 0.80 confidence threshold. Alpha diversity analysis was applied to assess the complexity of species diversity for a sample by using four indices: Chao1, Shannon, Simpson, ACE, and Observed species. These indices were calculated using QIIME (Version 1.8.0). Beta diversity analysis was used to evaluate differences in species complexity in the samples. Beta diversity was calculated using principal coordinates analysis (PCoA) and cluster analysis in the QIIME software (Version 1.8.0).

The data for body weight (BW), average daily gain (ADG), starter intake, rumen degradation rate of fiber, rumen metabolic phenotypes, digestive enzymatic activity, rumen histomorphology, microbial alpha diversity values, and the bacterial abundance were analyzed statistically using t-tests in the SPSS software (version 25.0; IBM Corp., Armonk, NY, United States). Spearman correlation coefficients were used to evaluate the relationships between the most abundant genera and rumen function-related parameters using the R software (version 4.1.1). Statistical significance was set at *p* < 0.05, and *p* < 0.001 indicated an extremely significant difference.

## 3. Results

### 3.1. Body weight, average daily gain, and starter intake

**Figure 2:**
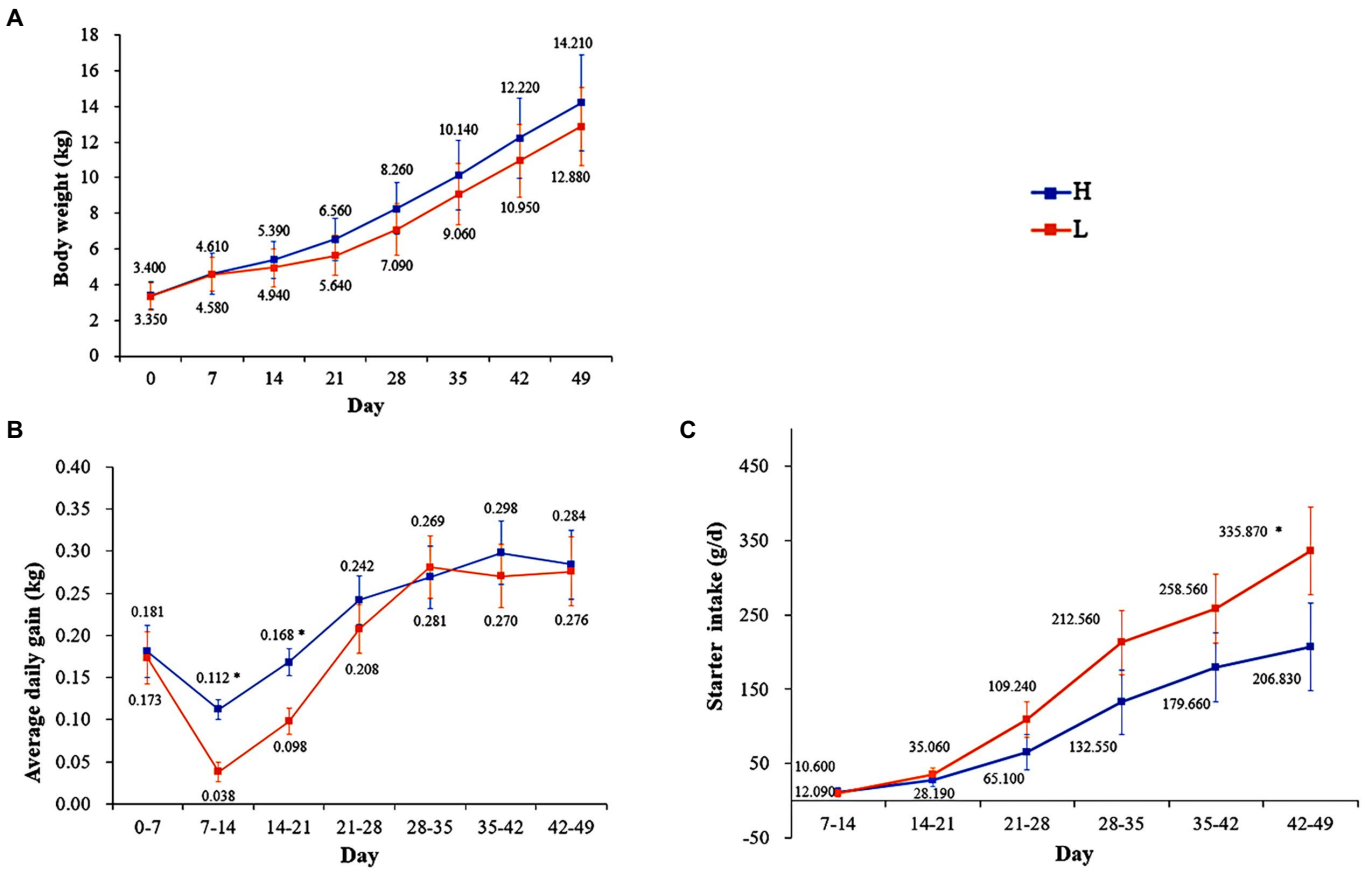
Effects of MR levels of on body weight **(A)**, average daily gain **(B)**, and starter intake **(C)**. H: high MR feeding level group, fed MR at 4% DM/kg of average body weight per day; L: low MR feeding level group, fed MR at 2% DM/kg of average body weight/d. * in the same column indicates a significant difference between two groups (*p* < 0.05).

There was no significant difference (*p* > 0.05) in BW between the groups; however, there was significant difference (*p* < 0.05) in the ADG at 7–14 and 14–21 day and starter intake (SI) at 42–49 day. In addition, the trend lines of BW and ADG were higher in the H group than in the L group, whereas the SI showed the opposite trend (Figure 2).

### 3.2. Ruminal fiber degradation rate

There were no significant differences in the NDF and ADF degradation rates between the groups (*p* > 0.05), nor were the contents of NDF and ADF different in the rumen digesta (Table 2).

**Table 2:** Effects of MR levels of on fiber degradation rate of lambs (%).

Index*	Groups		SEM	*p* Value
	L	H		
NDF	57.20	56.8	2.70	0.915
ADF	39.60	40.60	2.10	0.746
NDFD	53.70	53.20	3.20	0.913
ADFD	30.10	26.20	5.30	0.614

*NDF-rumen neutral detergent fiber content; ADF-rumen acid detergent fiber content; NDFD-degradation rate of NDF; and ADFD - degradation rate of ADF; H: high MR feeding level group, fed MR at 4% DM/kg of average body weight per day; L: low MR feeding level group, fed MR at 2% DM/kg of average body weight per day.

### 3.3. Rumen weight, pH, fermentation parameters, and enzyme activities

The rumen weight of the L group was significantly higher than that of the H group (*p* = 0.019); however, there was no significant effect on other indices in the rumen of the lambs (*p* > 0.05; Table 3).

**Table 3:** Effects of MR levels on the rumen weight, pH, fermentation parameters and enzyme activities of lambs.

Index	Groups		SEM	*p* Value
	L	H		
Rumen weight (g)	244.56^a^	160.49^b^	21.90	0.019
PH	6.56	6.70	0.07	0.207
Total VFA* (mmol/L)	65.39	51.17	8.14	0.240
Acetate (%)	50.58	52.89	1.57	0.319
Propionate (%)	32.89	29.52	1.47	0.132
Isobutyrate (%)	2.73	3.58	0.29	0.062
Butyrate (%)	6.64	6.75	0.60	0.898
Isovalerate (%)	4.29	4.88	0.60	0.500
Valerate (%)	2.86	2.38	0.36	0.369
Cellulase (U/mg protein)	72.58	114.53	33.17	0.389
Protease (U/mg protein)	0.54	0.52	0.17	0.939
Total nitrogen (mg/ml)	4724.96	5111.40	390.47	0.497
Ammonia nitrogen (mg/ml)	96.49	100.98	4.90	0.530
Urea nitrogen (mg/ml)	35.37	34.12	5.35	0.871

*The sum of all individual volatile fatty acids (VFA). In the same row, different small letter superscripts indicate a significant difference (*p* < 0.05), H: high MR feeding level group, fed MR at 4% DM/kg of average body weight per day; L: low MR feeding level group, fed MR at 2% DM/kg of average body weight per day.

### 3.4. Ruminal histomorphology

The rumen papillae length of the L group was significantly higher than that of the H group (*p* < 0.01); however, there were no significant effects on rumen papillae width and muscle layer thickness of the lambs (both *p* > 0.05; Table 4).

**Table 4:** Effects of MR levels on the rumen ruminal histomorphology of lambs.

Index	Groups		SEM	*p* Value
	L	H		
Papillae length	1964.36^a^	1032.59^b^	173.66	< 0.010
Papillae width	422.04	440.29	41.47	0.668
Muscle layer thickness	941.43	948.10	110.83	0.953

In the same row, different small letter superscripts indicate a significant difference (*p* < 0.05), H: high MR feeding level group, fed MR at 4% DM/kg of average body weight per day; L: low MR feeding level group, fed MR at 2% DM/kg of average body weight/d.

### 3.5. Ruminal microbiota diversity and community structure

The present study used 16S rRNA gene sequencing of rumen samples to compare the differences in the rumen microbiota between the H and L groups. The rarefaction curves showed that adequate sequencing depth was achieved, and the number of observed species was close to saturation (Supplementary Figure S1A). The total number of OTUs was 870, with 635 shared OTUs detectable across the groups; with 176 and 59 endemic species in the H and L groups, respectively (Supplementary Figure S1B). The Shannon (*p* = 0.042) and Chao1 (*p* = 0.047) indices significantly increased as the MR level increased from 2 to 4%; however, there was no significant change in the other indices (*p* > 0.05; Table 5).

**Table 5:** Rumen microbial richness and diversity indexes.

Index	Groups		SEM	*p* Value
	L	H		
Observed	374.57	436.43	25.58	0.113
Shannon	4.61^b^	5.11^a^	0.16	0.042
Simpson	0.89	0.91	0.01	0.245
Chao1	406.24^b^	476.61^a^	22.44	0.047
ACE	411.87	481.28	22.53	0.500

In the same row, different small letter superscripts indicate a significant difference (*p* < 0.05), H: high MR feeding level group, fed MR at 4% DM/kg of average body weight per day; L: low MR feeding level group, fed MR at 2% DM/kg of average body weight per day.

**Figure 3:**
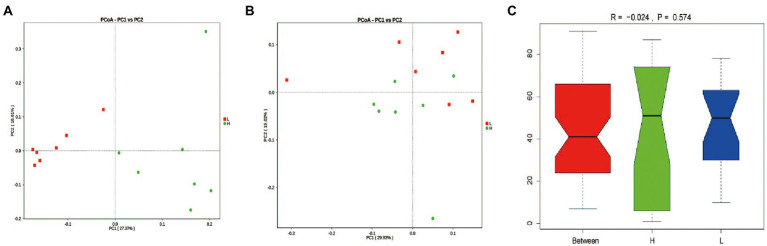
Rumen microbial OTU development. **(A)** Unweighted UniFrac and **(B)** Weighted UniFrac distances based on the relative abundance of microbial OTUs, **(C)** Bary-Curtis Anosim analysis. H: high MR feeding level group, fed MR at 4% DM/kg of average body weight per day; L: low MR feeding level group, fed MR at 2% DM/kg of average body weight per day.

Based on PCoA analysis (Figures 3A,B), there was clustering in the unweighted UniFrac measurements according to the L and H MR levels. However, the ANOSIM analysis based on Bary-Curtis showed an insignificant difference between groups (*p* = 0.574; Figure 3C).

**Figure 4:**
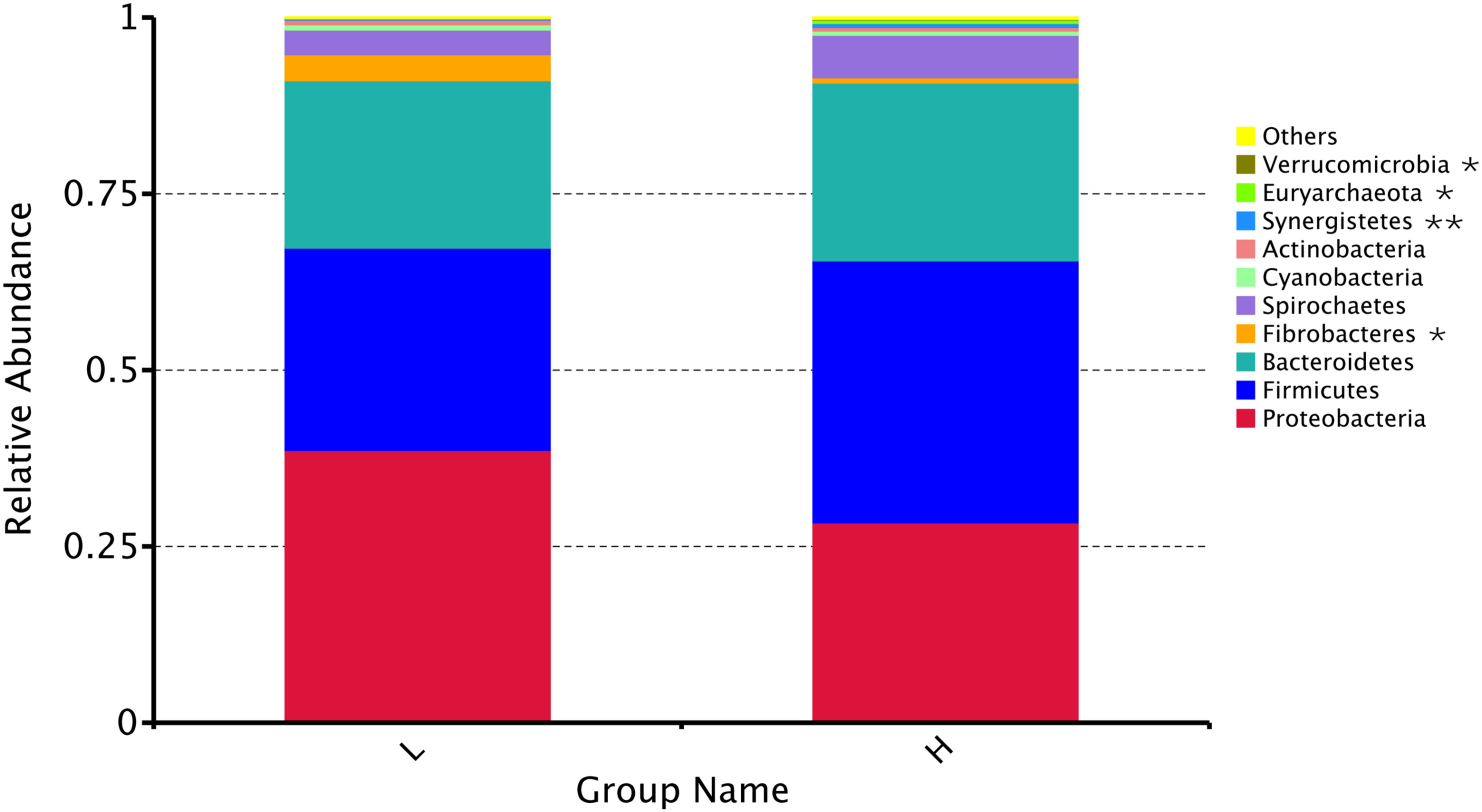
Relative abundance of the top 10 rumen microbial compositions at the phylum level. H: high MR feeding level group, fed MR at 4% DM/kg of average body weight per day; L: low MR feeding level group, fed MR at 2% DM/kg of average body weight per day. * indicates a significant difference (*p* < 0.05), and ** indicates an extremely significant difference (*p* < 0.01) between two groups.

The dominant flora (Figure 4) in the rumen (relative level > 5%) were Proteobacteria, Firmicutes, and Bacteroidetes at the phylum level. The relative abundance of Fibrobacteres (*p* = 0.033) decreased significantly, and the relative abundance of Synergistetes (*p* = 0.005), Euryarchaeota (*p* = 0.012), and Verrucomicrobia (*p* = 0.038) increased significantly with the increasing MR feeding level.

The dominant flora (Table 6) in the rumen (relative level > 5%) in group L were *Succinivibrionaceae_UCG-001*, *Succinivibrio*, *Prevotella_7*, *Prevotella_1*, *Oribacterium*, and *Lachnospiraceae_NK3A20_group*, and the dominant flora in the rumen in group H were *Succinivibrionaceae_UCG-001*, *Succinivibrio*, *Prevotella_7*, *Prevotella_1*, and *Oribacterium*. Among them, *Succinivibrionaceae_UCG-001* was the first largest flora, accounting for 15.21 and 18.24% in L and H groups, respectively, and *Succinivibrio* is the second largest flora, accounting for 8.88 and 14.37% in L and H groups, respectively. The relative abundance of *Lachnospiraceae_NK3A20_group* (*p* = 0.007), *Succiniclasticum* (*p* = 0.035), and *Ruminococcus_1* (*p* = 0.034) was significantly higher in the L group, and the relative abundance of *Ruminococcaceae_UCG-014*, *Moryella*, and *Eubacterium_nodatum_group* in the L group exhibited tended to be higher than that in the H group (*p <* 0.1).

**Table 6:** Genus-level taxonomic composition of the top 30 rumen microbial communities (%).

Genus	Groups		SEM	*p* Value
	L	H		
*Succinivibrionaceae_UCG-001*	15.21	18.24	2.99	0.632
*Succinivibrio*	8.88	14.37	3.31	0.430
*Prevotella_7*	6.03	10.29	1.74	0.234
*Prevotella_1*	9.48	5.40	1.78	0.267
*Oribacterium*	7.15	7.40	1.18	0.922
*Lachnospiraceae_NK3A20_group*	5.56^a^	3.05^b^	0.51	0.007
*Ruminobacter*	3.67	2.85	2.10	0.855
*Succiniclasticum*	3.98^a^	1.97^b^	0.56	0.035
*Treponema_2*	3.40	1.88	0.78	0.352
*Fibrobacter*	0.74	3.70	1.29	0.268
*Sphaerochaeta*	2.64	1.61	0.42	0.237
*Ruminococcus_1*	2.97^a^	1.15^b^	0.44	0.034
*Succinivibrionaceae_UCG-002*	0.02	2.69	1.30	0.325
*Megasphaera*	0.76	1.93	0.44	0.196
*Dialister*	0.24	2.18	0.86	0.274
*Ruminococcaceae_UCG-014*	1.65	0.72	0.25	0.065
*Selenomonas*	0.91	1.15	0.22	0.600
*Rikenellaceae_RC9_gut_group*	0.79	1.23	0.41	0.611
*Mitsuokella*	0.44	1.37	0.37	0.228
*Roseburia*	0.77	0.93	0.16	0.636
*Syntrophococcus*	0.93	0.56	0.13	0.154
*Ruminococcaceae_NK4A214_group*	1.01	0.41	0.20	0.139
*Sharpea*	0.72	0.54	0.22	0.701
*Ruminococcus_2*	0.61	0.40	0.11	0.374
*Moryella*	0.72	0.16	0.16	0.074
*Eubacterium_nodatum_group*	0.70	0.16	0.15	0.076
*Prevotellaceae_UCG-001*	0.37	0.38	0.08	0.956
*Lachnospiraceae_XPB1014_group*	0.61	0.02	0.27	0.281
*Pyramidobacter*	0.46	0.16	0.10	0.131
*Erysipelotrichaceae_UCG-004*	0.05	0.55	0.22	0.282
Other	0.07^a^	0.04^b^	0.01	0.012

In the same row, different small letter superscripts indicate a significant difference (*P* < 0.05), and H-high MR feeding level group and L-low MR feeding level group, H: high MR feeding level group, fed MR at 4% DM/kg of average body weight per day; L: low MR feeding level group, fed MR at 2% DM/kg of average body weight per day.

### 3.6. Correlation analysis of rumen weight, pH, fermentation parameters, enzyme activity, ruminal histomorphology and microorganisms

**Figure 5:**
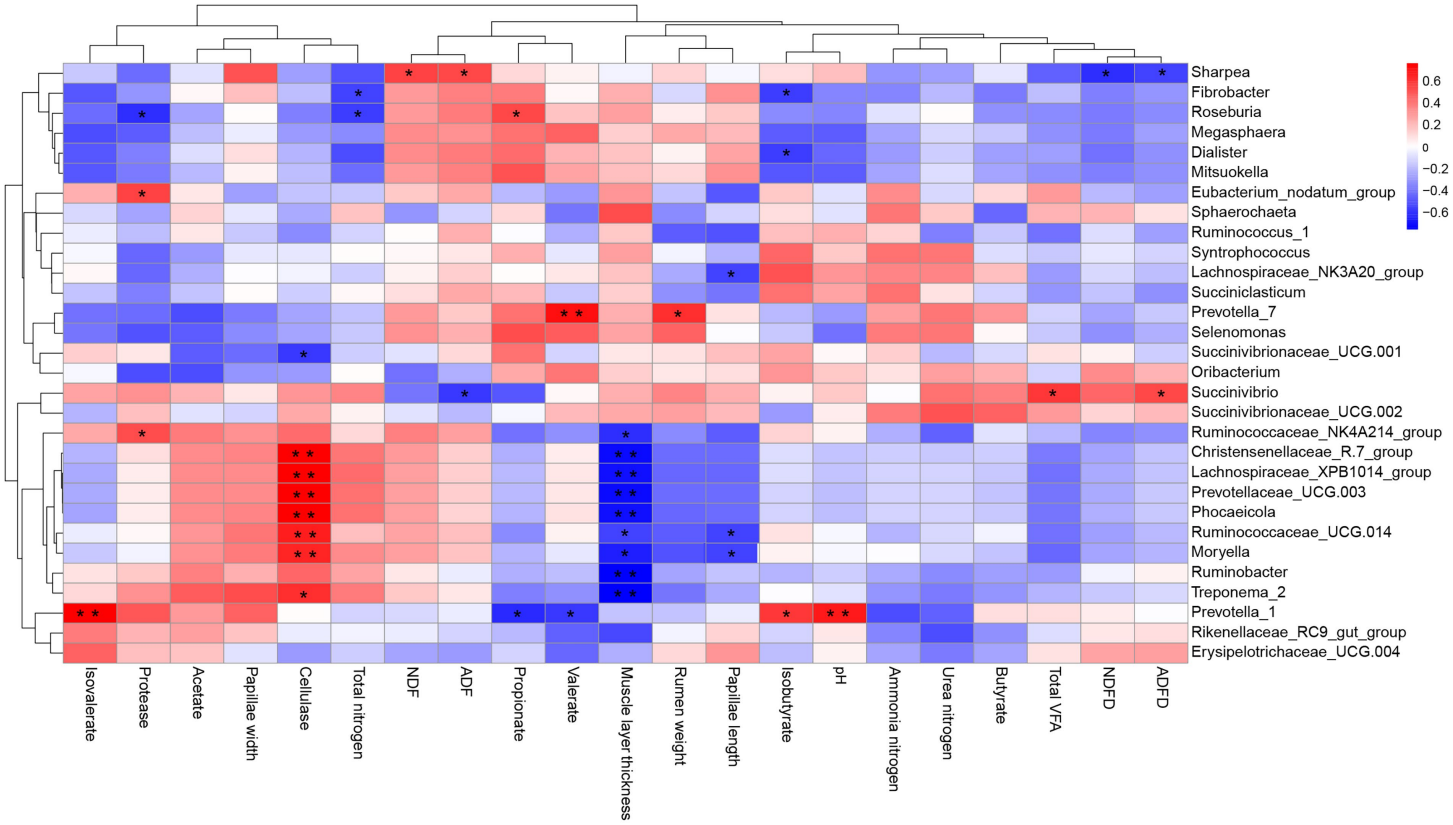
Correlation between the top 30 genus-level taxonomic compositions and rumen weight, pH, fermentation parameters, and enzyme activity. *p < 0.05, **p < 0.01.

Correlation analyses of the relative abundances of the top 30 genus-level taxonomic composition and rumen weight, pH, fermentation parameters, enzyme activity, and rumen fiber degradation were performed (Figure 5). The results showed that the relative abundance of *Christensenellaceae_R-7_group* (*p* = 0.002), *Lachnospiraceae_XPB1014_group* (*p* = 0.001), *Prevotellaceae_UCG-003* (*p* = 0.01), *Phocaeicola* (*p* = 0.001), *Ruminococcaceae_UCG-014* (*p* = 0.008), *Moryella* (*p* = 0.01), and *Treponema_2* (*p* = 0.015) correlated positively with cellulase activity, whereas the abundance of *Succinivibrionaceae_UCG-001* (*p* = 0.03) correlated negatively with cellulase activity. The relative abundance of *Ruminococcaceae_NK4A214_group* (*p* = 0.023), *Christensenellaceae_R-7_group* (*p* = 0.004), *Lachnospiraceae_XPB1014_group* (*p* = 0.005), *Prevotellaceae_UCG-003* (*p* = 0.004), *Phocaeicola* (*p* = 0.005), *Ruminococcaceae_UCG-014* (*p* = 0.042), *Moryella* (*p* = 0.01), and *Treponema_2* (*p* = 0.003) *Ruminobacter* (*p* = 0.002) correlated negatively with muscle layer thickness. The relative abundance of *Lachnospiraceae_NK3A20_group* (*p* = 0.047), *Ruminococcaceae_UCG.014* (*p* = 0.042), *Moryella* (*p* = 0.041) correlated negatively with papillae length. *Succinivibrio* correlated positively with the ADF degradation rate (*p* = 0.039) and the total VFA concentration (*p* = 0.024), whereas the NDF (*p* = 0.02) and ADF (*p* = 0.042) degradation rates correlated positively with *Sharpea*. The valerate (*p* = 0.004) concentration and rumen weight (*p* = 0.016) correlated positively with *Prevotella_7*. The propionate concentration correlated negatively with *Prevotella_1* (*p* < 0.017) and *Roseburia* (*p* = 0.041). The isovalerate concentration (*p* = 0.001), isobutyrate concentration (*p* = 0.023), and pH (*p* = 0.07) correlated positively with *Prevotella_1*. Total nitrogen correlated negatively correlated with *Fibrobacter* and *Roseburia* (both *p* = 0.04). Protease correlated positively with *Eubacterium_nodatum_group* (*p* = 0.035) and *Ruminococcaceae_NK4A214_*group (*p* = 0.049), whereas it correlated negatively with *Roseburia* (*p* = 0.023).

## 4. Discussion

To obtain a higher price for lambs and more income from a flock, it is essential to have the minimum loss of lambs and optimum growth during the neonatal phase (Poonia et al., 2015). Meanwhile, appropriate feeding strategies to promote rumen development and microbiota establishment might be beneficial to the performance of lambs in the later period. Despite the accumulated knowledge regarding nutritional regulatory strategies during the early life of ruminants, the effects of the MR feeding level on rumen development, microbial colonization, and the regulatory mechanisms of host-microbial interactions in pre-ruminants are largely unknown.

Up to 3 weeks old, the growth performance of lambs was significantly improved by the higher level of MR feeding, although the low-level feeding group had a higher starter feed intake. The intake of solid feed in lambs before 3 weeks old is low, and the digestion and utilization efficiency of plant-based feed was much lower than that of MR (Li et al., 2022). Thus, at this stage, milk or MR provides most of the nutrients needed for growth and development, and the better bioavailability of protein and energy along with minerals, enzymes, and growth factors results in increased weight gains (Lee et al., 2009). Studies have also shown that high levels of MR can increase the ADG of lambs before weaning (Zhang et al., 2019). After the were 4 weeks old, with the rapid improvement of starter intake and digestive function, the effect of the MR feeding level on daily gain was no longer significant, indicating that the development of digestive function and the increase of starter intake are very important for the transition from liquid milk to plant feed in young lambs.

It should be noted that although high levels of MR feeding improved the growth performance of early lambs, it had adverse effects on rumen development. In the present study, the rumen weight and papillae length at 49 days old was significantly lower in the high feeding level group, and the lower intake of starter was the main factor limiting rumen development. Studies have shown that feeding large volumes of milk delays solid feed intake, which might compromise rumen development before and during weaning (Jasper and Weary, 2002). An adequate starter intake can stimulate the development of the rumen, which is necessary to establish the rumen microbiota (Khan et al., 2016). In early lambs, MR bypasses the rumen to the abomasum (Zhang et al., 2019), therefore, the solid feed that enters the rumen is the decisive factor for rumen fermentation parameters. Interestingly, we did not find any differences in the main rumen fermentation parameters, enzyme activities, and fiber degradation rates between the groups in the present study. Considering that the two groups of lambs have the same diet composition, these results indicated that the composition of the starter diet was more important for rumen fermentation function than the starter intake.

Interestingly, we found that the MR feeding level had a profound influence on the rumen microbiota, which was reflected in the α-diversity, β-diversity, and taxa abundance. In this study, despite the increasing of starter intake, the Shannon and Chao1 indices of the rumen microbiota decreased in L group. Although highly diverse microbiota is generally considered beneficial for host health and is regarded as a sign of a mature gut (Konopka, 2009; Le Chatelier et al., 2013), many studies have indicated that early starter intake decreased the ruminal bacterial diversity (Meale et al., 2016; Wang et al., 2016; Li et al., 2020). These findings were consistent with the results obtained from this study. We found that many bacteria genera and species disappeared in L group. The main reason may be that the increased starter intake promotes the establishment of predominant microflora and the depletion of transient bacterial species and genera. With the increase of plant-based feed intake, carbohydrate degrading bacteria occupied the widest niche, which suppressed the colonization of foreign flora, and most of the ‘disappeared’ bacteria are aerobic bacteria or bacteria without fermentation function (Wang et al., 2016). Besides, on average, the phyla Proteobacteria, Firmicutes, and Bacteroidetes were predominant in all samples. These findings agreed with previous studies on the ruminal microbiota (Morgavi et al., 2015). Proteobacteria are a large group of bacteria that ferment carbohydrates to ethanol, playing an important role in rumen metabolism, such as the formation and fermentation of biofilms (Zeng et al., 2017). Firmicutes represent the core bacterial component that is predominant within the rumen, mainly comprising diverse fibrolytic and cellulolytic bacterial genera. Bacteroidetes express relatively large numbers of genes encoding carbohydrate-active enzymes; thus, promoting the breakdown of structural polysaccharides in the rumen and also fermenting amino acids into acetate (Zhang Y. K. et al., 2021). Notably, the high MR feeding level decreased the relative abundance of Fibrobacteres, which were reported as major bacterial degraders of lignocellulosic material in the herbivore gut (Ransom-Jones et al., 2012). High MR feeding level decreased the starter intake, and the reduced available substrates may be the main reason for the reduction of lignocellulosic degrading bacteria. Besides, the high MR feeding level increased the relative abundance of Synergistetes, Euryarchaeota, and Verrucomicrobia, which have been reported to be associated with inflammation in the gastrointestinal epithelium (Forterre et al., 2014; Sichert et al., 2020; McCracken and Nathalia Garcia, 2021), suggesting that excessive feeding of MR might have adverse effects on gastrointestinal health by altering the microbiota.

In particular, we found that the MR feeding level affected the abundance of many carbohydrate-degrading bacteria. The abundance of *Lachnospiraceae_NK3A20_group*, *Succiniclasticum*, *Ruminococcus_1*, *Ruminococcaceae_UCG-014*, and *Moryella* decreased with the increasing MR level. *Lachnospiraceae* ferment diverse plant polysaccharides to short-chain fatty acids and alcohols (Boutard et al., 2014). *Succiniclasticum*, which ferment succinate quantitatively to propionate, were isolated from a high dilution of rumen ingesta obtained from a dairy cow fed a production diet containing grass silage as the main roughage source (Van Gylswyk, 1995). *Ruminococcus_1* plays an important role in plant fiber degradation (Devillard et al., 2004). *Moryella* has been reported to be associated with cellulose or its metabolites (Tian et al., 2019). A key deterministic factor framing the process of microbial succession is diet, especially because the abundance of certain bacteria changes in response to a fiber-based diet (Furman et al., 2020). The pre-ruminant rumen microbiota is highly active and ready to ferment a solid diet from the first week of life (Malmuthuge et al., 2019), and the increased abundance of certain important fiber degrading bacteria might be an adaptive mechanism of the rumen microbiota in response to increased solid diet intake. Typically, under *ad libitum* feed intake, as the starter intake increases, the rate of digesta passage from the rumen increases and nutrient digestibility decreases (Bourquin et al., 1990). In this study, the starter intake was significantly lower in the high MR feeding level group; however, the rumen degradation rates of NDF and ADF did not increased, which mighty be related to the decreased abundance of these carbohydrate-degrading bacteria.

We further analyzed the relationship between rumen fermentation parameters and bacterial abundance, and found that many bacterial genera with significant differences, such as *Ruminococcaceae_UCG-014* and *Moryella*, correlated significantly and positively with rumen cellulase activity, and *Succinivibrio* correlated positively with rumen ADF degradation rate and the total VFA concentration. Considering the small sample size and the vast differences in microbiome composition between the different groups, we cannot infer specific links through correlation due to large fluctuations in the microbiome between groups. This would likely yield inaccurate correlations due to strong habitat filtering (Berry and Widder, 2014) and the heteroscedasticity of the data. Although the correlation relationship does not necessarily indicate a direct causal effect, the observed multiple significant correlations between relative abundances of microbial taxa and rumen fermentation parameters provide some insight into potential host-microbiotic interactions in the rumen, suggesting that changes in the microbiota, especially the abundance of carbohydrate-degrading bacteria, may be the mechanisms for the rumen to adapt to changes in food structure and starter intake and can affect rumen function. There has been a study indicating that VFAs produced by the early microbiome were associated with the rumen tissue metabolism and the development of the epithelium with the host transcriptome and microRNAome (Malmuthuge et al., 2019). However, further studies are needed to determine the long-term consequences of the adverse effects of a high MR feeding level on rumen weight and the microbiota of lambs. A suitable balance between promoting rumen development and enhancing weight gain in early stage needs to be determined by selecting appropriate levels of MR feeding.

## 5. Conclusion

Increasing the feeding level of MR from 2 to 4% of the body weight of lambs significantly increased the weight gain, but decreased the intake of starter and had adverse effects on rumen development. A high MR feeding level increased the rumen microbial diversity, but decreased the abundance of many rumen carbohydrate catabolizing bacteria. This study provides new insights into the regulation of early rumen development (function, morphology, and microbial colonization) of lambs. The results suggests that the adverse effect of excessive MR feeding on rumen development and the microbiota should not be ignored; and a suitable balance between promoting rumen development and enhancing weight gain in the early stage needs to be identified by selecting appropriate levels of MR feeding.

## Data availability statement

The datasets presented in this study can be found in online repositories. The names of the repository/repositories and accession number(s) can be found at: https://www.ncbi.nlm.nih.gov/, PRJNA890401.

## Ethics statement

The animal study was reviewed and approved by the Gansu Agricultural University’s Academic Committee and the National Natural Science Foundation of China (Approval no. 31760682) according to guidelines established by the Biological Studies Animal Care and Use Committee of Gansu Province.

## Author contributions

YH, GW, and QZ: methodology, investigation, and writing-original draft. ZC, XZ, XW, and DZ: validation and visualization. CL: funding acquisition. WW: supervision and project administration. PC, ZM, and CL: writing-review and editing. All authors contributed to the article and approved the submitted version.

## Funding

This work was supported by the National Natural Science Foundation of China under grant no. 31760682 and Discipline Team Project of Gansu Agricultural University under grant no. GAU-XKTD-2022-20.

## Conflict of interest

The authors declare that the research was conducted in the absence of any commercial or financial relationships that could be construed as a potential conflict of interest.

## Publisher’s note

All claims expressed in this article are solely those of the authors and do not necessarily represent those of their affiliated organizations, or those of the publisher, the editors and the reviewers. Any product that may be evaluated in this article, or claim that may be made by its manufacturer, is not guaranteed or endorsed by the publisher.

## Footnotes

^1^ http://ccb.jhu.edu/software/FLASH/

^2^ http://drive5.com/uparse/

^3^ http://www.arb-silva.de/
