# Supplementary material for: Effects of milk replacer feeding level on growth performance, rumen development and the ruminal bacterial community in lambs
Source: Front Microbiol. 2023 Jan 10;13:1069964. doi: 10.3389/fmicb.2022.1069964 (PMC9871810; doi:10.3389/fmicb.2022.1069964)
Supplement: Supplementary file 1 [file Table_1.DOCX]

Supplementary Material

Effects of milk replacer feeding level on growth performance, rumen development and the ruminal bacterial community in lambs

Yongliang Huang^1^, Guoxiu Wang^1^, Qian Zhang^2^, Zhanyu Chen^1^, Chong Li^1, *^, Weimin Wang^1, 2^, Xiaoxue Zhang^1^, Xiaojuan Wang^1^, Deyin Zhang^2^, Panpan Cui^1^, Zongwu Ma^1^

*** Correspondence:** Chong Li: [lichong@gsau.edu.cn](mailto:lichong@gsau.edu.cn)

# Supplementary Figures

A
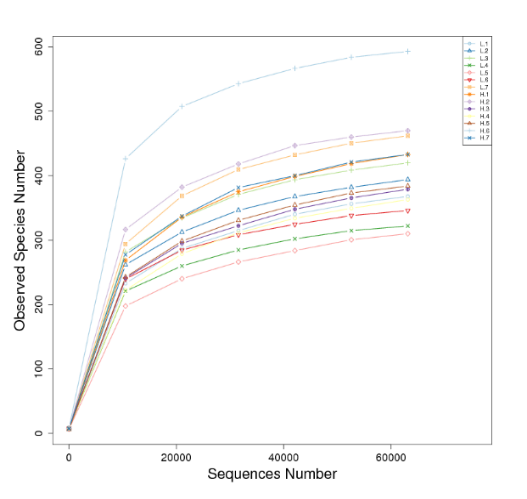
B
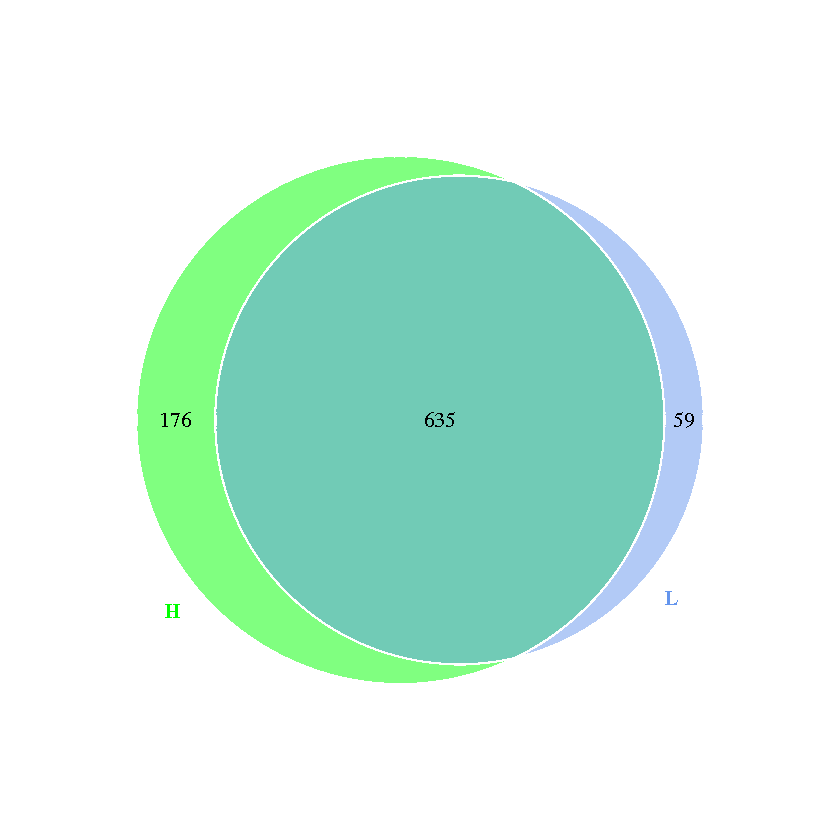


**Figure S1.** **(A)** Observed indices were used to assess the depth of coverage for each sample, each sample was distinguished by different colored lines. **(B)** Venn diagrams for rumen microbial OTU compositions. H: high MR feeding level group, fed MR at 4% DM/kg of average body weight per day; L: low MR feeding level group, fed MR at 2% DM/kg of average body weight/d.
